# Effects of changing modulation and pitch parameters on tomotherapy delivery quality assurance plans

**DOI:** 10.1120/jacmp.v16i5.5282

**Published:** 2015-09-08

**Authors:** Diana Binny, Craig M. Lancaster, Selina Harris, Steven R. Sylvander

**Affiliations:** ^1^ Cancer Care Services Royal Brisbane and Women's Hospital QLD Australia; ^2^ Science and Engineering Faculty Queensland University of Technology QLD Australia

**Keywords:** modulation factor, tomotherapy DQA plans, leaf open times, dose‐volume histograms

## Abstract

This study was aimed at investigating delivery quality assurance (DQA) discrepancies observed for helical tomotherapy plans. A selection of tomotherapy plans that initially failed the DQA process was chosen for this investigation. These plans failed the fluence analysis as assessed using gamma criteria (3%, 3 mm) with radiographic film. Each of these plans was modified (keeping the planning constraints the same), beamlets rebatched and reoptimized. By increasing and decreasing the modulation factor, the fluence in a circumferential plane as measured with a diode array was assessed. A subset of these plans was investigated using varied pitch values. Metrics for each plan that were examined were point doses, fluences, leaf opening times, planned leaf sinograms, and uniformity indices. In order to ensure that the treatment constraints remained the same, the dose‐volume histograms (DVHs) of all the modulated plans were compared to the original plan. It was observed that a large increase in the modulation factor did not significantly improve DVH uniformity, but reduced the gamma analysis pass rate. This also increased the treatment delivery time by slowing down the gantry rotation speed which then increases the maximum to mean non‐zero leaf open time ratio. Increasing and decreasing the pitch value did not substantially change treatment time, but the delivery accuracy was adversely affected. This may be due to many other factors, such as the complexity of the treatment plan and site. Patient sites included in this study were head and neck, right breast, prostate, abdomen, adrenal, and brain. The impact of leaf timing inaccuracies on plans was greater with higher modulation factors. Point‐dose measurements were seen to be less susceptible to changes in pitch and modulation factors. The initial modulation factor used by the optimizer, such that the TPS generated ‘actual’ modulation factor within the range of 1.4 to 2.5, resulted in an improved deliverable plan.

PACS number: 87.55.‐x, 87.55.Qr, 87.55.D‐

## I. INTRODUCTION

Helical tomotherapy has proven to be an improved form of IMRT in its ability to conform planned dose distributions to clinically designated target volumes and it has been shown previously that, by modifying planning parameters, treatment outcomes can be improved.^1^, ^2^ TomoTherapy Hi·Art II system (Accuray, Sunnyvale, CA) uses a 6 MV linear accelerator (linac) mounted on a slip ring gantry with intensity‐modulated fan beams delivered helically. The tomotherapy system consists of a fast binary MLC with a tenth of the leakage. These binary leaves move at 100 times the speed compared to conventional MLCs with an average open to close time of 20 ms. The megavoltage CT (MVCT) system is used for pretreatment setup verification, delivery verification, dose reconstruction, and also for machine quality assurance purposes.^(3)^

Planning studies have demonstrated the dosimetric advantages of using helical tomotherapy for sites like breast, prostate, brain, and head and neck over conformal treatments.^4^, ^5^, ^6^, ^7^ Optimal plan results depend on the capability of the optimizer and the use of appropriate planning parameters to achieve desired constraints. In this study, planned parameters of pitch and modulation factor have been modified to achieve the best plan outcome assessed by comparing dose‐volume histograms (DVHs), measured fluences, point dose, leaf open times (LOTs), planned sinograms, and treatment delivery times for all plans before and after the changes were applied. Pitch is the couch travel distance for a complete gantry rotation with respect to the beam width on the axis of rotation. The modulation factor (MF) is a number that reflects the trade‐off between plan efficiency and freedom of the optimizer to vary beamlet intensities to achieve planning goals. This is only an upper limit in the planning system from which the beam intensity for all projections is calculated by dividing the maximum leaf open time by the average leaf open times of all non‐zero leaf open time.^(1)^ At the RBWH, it was observed that certain plans failed patient specific DQAs despite stable daily machine outputs and hence this study was undertaken to investigate these failed planned fluences by changing planning parameters.

According to vendor recommendations, increasing modulation factor may help improve the DVHs; however, the deliverability of a more complex plan was investigated in this study. Once the gantry period reaches its minimum permissible value of 12 s, the gantry period can no longer compensate for further decrease in the pitch, making the plan inefficient. This was investigated by increasing and decreasing pitch parameters for a subset of plans that displayed a tendency to change rapidly with change in modulation factors. The results were compared to the original plan. The TomoTherapy Hi·Art II treatment planning system (TPS) v.4.2.2 uses a collapsed cone (CC) algorithm to calculate dose to medium using a calculation grid of $0.273\;{\text{cm}}^{2}$. The delivery quality assurance (DQA) procedure followed compared the planned point dose with the cylindrical tomotherapy ‘cheese’ phantom measured dose using an Exradin A1SL chamber (Standard Imaging, Middleton, WI) in a solid water cylinder with radiographic film (EDR2, Carestream Health Inc, Rochester, NY) for fluence measurements.^8^, ^9^ Planning strategies, like increasing and decreasing modulation and pitch parameters, were carried out on the failed plans and the replanned fluences were verified using the ArcCHECK diode array (Sun Nuclear Corporation (SNC), Melbourne, FL).

## II. MATERIALS AND METHODS

### A. Study plans and IMRT equipment

Tomotherapy DQA involves patient‐specific QA for every plan approved by the physician, using the planned and delivered gamma analysis of the relative dose distribution and point‐dose comparisons. The criterion for acceptable calculation performance is defined as the tolerance of dose and is set at ±3% for a dose or to distance to agreement (DTA) of 3 mm. The action level is specified at 90% and above for plans to meet the pass criteria if using film. The ArcCHECK used a gamma dose criterion of ±3% and a DTA of 2 mm to accommodate the decreased resolution when compared to film with an action level of 95% and above. The approved plan was first calculated on the cheese phantom and then measured using an EDR2 film placed in the coronal plane of the cylindrical cheese phantom to provide full scatter conditions. An A1SL chamber is placed directly below this film plane to measure the point dose in the PTV. Using this method, a relative dose profile and an absolute point‐dose measurement are acquired, which are compared to their corresponding CC‐calculated profile and point dose. Plans that were chosen for this study failed this DQA procedure during their fluence analysis; however, point doses were still within their approved tolerances (±3%). These failed plans were then verified using the ArcCHECK phantom that has 1386 diode detectors arranged helically with 10 mm spacing. The center of this phantom is designed to accommodate accessories like ion chambers and heterogeneous materials for dose studies.

Patient plans that had failed the initial analysis process were replanned using increasing and decreasing modulation and pitch parameters. These modified plans were verified using the ArcCHECK. The A1SL chamber previously used in the cheese phantom was placed in a chamber holder that is fitted in a polymethyl methacrylate (PMMA) cylinder inside the ArcCHECK phantom for point‐dose measurements, and the diode array was used for fluence measurements. The dose and plan files were exported from the TPS and independently compared with the measurements using the SNC software.

The pitch values for all plans were chosen according to the rule

(1) $$Pitch=\frac{0.86}{N}\;\text{for N}=1,2,3\ldots$$

where *N* is an integer and *0.86* is an empirical factor that accounts for the beam junctioning of off‐axis profiles which differ from the axial profiles with depth.^(11)^ This formula showed the use of a good pitch to minimize thread effects on large patient plans as increasing the pitch value results in loss of longitudinal resolution in the dose distribution. Three patient plans used a lower or a higher pitch to demonstrate differences in fluences, planned leaf sinograms, and point doses for that particular set parameter. Planning modulation factors were increased using arbitrary values; 0.5, 1, 2, and maximum value of 5 and decreased by 0.5 and 1. Three plans were reoptimized using pitch values of 0.430 or 0.287.

A subset of plans was selected for the analysis using changed pitch values on the basis that the plans either showed no significant change in their DVHs with change in modulation factor or required reoptimization. Table 1 shows initial patient plan parameters that were chosen for the replan using ArcCHECK. The term “Pat” is used in the tables to indicate Patient ID and “$\%{\text{diff}}_{\text{IC}}$” refers to the percentage difference in the ion chamber readings when compared to planned dose.

**Table 1:** Summary of initial patient plan parameters

*Treatment Site*	*Pat*.	*Planning MF*	*Actual MF*	*Pitch*	*Field Width*	*Coronal Film Gamma Pass Rate*	$\%{\text{diff}}_{\text{IC}}$
RT Breast	A	2.9	1.9	0.287	5.0	79	0.9
Prostate	B	2.2	1.9	0.430	2.5	85	1.5
Adrenal	C	2.0	1.5	0.430	2.5	90	1.4
Abdomen	D	2.5	2.0	0.287	2.5	76	1.6
Brain	E	2.5	2.1	0.287	1.0	82	0.5
Head and Neck	F	2.2	2.0	0.430	2.5	90	2.6

### B. DVH analysis

To ensure the replans were clinically acceptable, all the planning target volumes (PTVs), homogeneity indices (HI), uniformity indices (UI), and doses to the organ at risk (OAR) were checked against the initial approved plan. The effects of changing modulation factors on these replans were also observed.

Uniformity index (UI) was calculated using the formula D5/D95, where D5 and D95 are the dose to 5% and 95% of target volume. Homogeneity index (HI) was calculated using $(D2-D98)/D_{p}$, where D2 and D98 are the dose to 2% and 98% of the target volume and $D_{p}$ is the target prescription dose used during optimization.^12^, ^13^, ^14^

Organ at risk (OAR) volumes were assessed using QUANTEC protocols.^(12)^ V25<50% refers to the criterion that the volume of OAR that receives 25 Gy or more should be less than 50% of the total volume. Plans that failed dose volume constraints during modulation factor modifications were taken note of and the gamma indices for those plans were not included in the results as they were not regarded as to be clinically acceptable.

### C. Point dose measurements and fluence comparisons

In order to compare the TPS calculated dose to the measured dose to medium in PMMA the mass energy absorption coefficient ratio for air to PMMA of 1.031 was applied to dose to medium.^(15)^ The ArcCHECK virtual CT imported for planning used a uniform physical density of $1.152\;g{\text{cm}}^{-3}$ (PMMA). All measured fluences for optimized plans using increasing and decreasing modulation and pitch factors were compared to their corresponding planned fluence and point dose. A threshold of 2%/3mm was applied when using ArcCHECK (gamma pass>95%), while the coronal film used a 3%/3mm tolerance (gamma pass rate>90%).

### D. Planned leaf sinograms and gantry period

Planned leaf sinograms and LOTs for all plans were used to compare with their corresponding fluence and point‐dose analysis to predict a plan's pass rate prior to patient specific checks. Planned leaf sinograms provided an indication of the projection intensity per leaf, which can suggest the delivery time and leaf speed. To visualize the planned leaf sinograms all patient plans were read into the vendor's MATLAB program (The MathWorks, Natick, MA, USA) borrowed during the study and the projection versus leaf number for each plan was acquired. Gantry period of rotation for all plans were also noted which were a contributing factor to beam‐on times.

## III. RESULTS

### A. Actual modulation factors

Upper and lower limits for modulation factors were used for optimization in this investigation. From 2, 3 it can be seen that the planning modulation factor only sets an upper limit to avoid unwanted modulation of the plan, thereby giving the planner control over the treatment times and DVHs. In case of Patient D, the small fractional increment of 0.5 did not affect the actual modulation factor as the optimal solution had already been achieved in this case.

**Table 2:** Planned modulation factors

*Treatment Site*	*Pat*.	*MF*	MF+0.5	MF+1	MF+2	MF=5	MF−0.5	MF−1
RT Breast	A	2.9	3.4	3.9	4.9	5.0	2.4	1.9
Prostate	B	2.2	2.7	3.2	4.2	5.0	1.7	1.2
Adrenal	C	2.0	2.5	3.0	4.0	5.0	1.5	1.0
Abdomen	D	2.5	3.0	3.5	4.5	5.0	2.0	1.5
Brain	E	2.5	3.0	3.5	4.5	5.0	2.0	1.5
Head and Neck	F	2.2	2.7	3.2	4.2	5.0	1.7	1.2

**Table 3:** Actual modulation factors used by TPS

*Treatment Site*	*Pat*.	*MF*	MF+0.5	MF+1	MF+2	MF=5	MF−0.5	MF−1
RT Breast	A	1.9	2.2	2.5	3.2	3.2	2.4	1.9
Prostate	B	1.9	2.2	2.7	3.8	4.2	1.5	1.1
Adrenal	C	1.5	1.9	2.2	2.9	3.7	1.2	1.0
Abdomen	D	2.0	2.0	2.7	3.4	3.9	1.9	1.3
Brain	E	2.1	2.5	2.9	3.7	4.1	1.7	1.4
Head and Neck	F	2.0	2.4	2.9	3.8	4.8	1.5	1.1

### B. DVH Analysis

**Figure 1:**
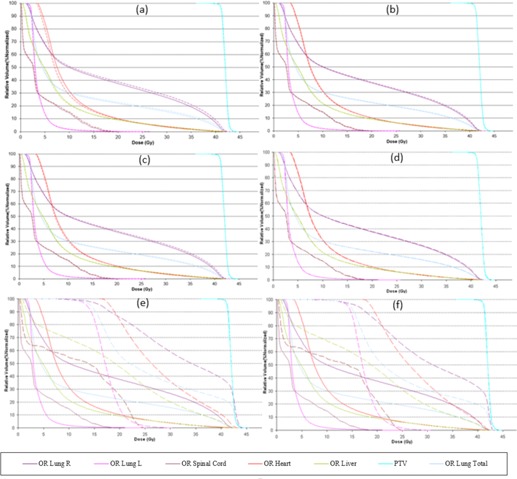
Patient ART breast plan. Dose‐volume histograms for the initial MF (solid lines) compared with (a) MF+0.5. (b) MF+1, (c) MF+2, (d) MF=5, (e) MF−0.5, and (f) MF−1 (dashed lines).

It was observed that, in most cases, increasing the modulation factor improved target volume coverage and maintained a good OAR dose‐to‐volume limit; however, this was not seen for cases when the modulation factors were reduced. As shown in Table 4 and Figs. 1 (e) and (f), the optimizer in the case of reduced modulation factors can cause the dose constraints to be out of tolerance delivering very high doses to OAR, as per published data^12^, ^13^, ^14^ and compromising the PTV coverage. This is due to the reduced treatment time impact where the leaf open times are varied very rapidly resulting in a nonclinically acceptable plan.

Other factors, like complexity of the treatment area, can also play a significant role in the optimization and, even with a good pitch value or a modulation factor, it is possible to achieve an unacceptable distribution. A planner may also be able to improve this nonuniformity by making changes in the pitch value; however, modifications in the modulation factor are also made to ensure optimal gantry rotation period is achieved. DVHs, target, and OAR indices are shown for all patient plans (1, 2, 3, 4, 5, 6 and 4, 5, 6, 7, 8, 9). Solid lines in the DVHs represent structures in the initial modulation factor (MF) plan, while the dashed lines represent the structures for the modified MF. Changes in pitch values have only been tested on the initial plan, while all other plans have made use of the initially planned pitch.

Patient A plan is shown in Fig. 1 and Table 4

**Figure 2:**
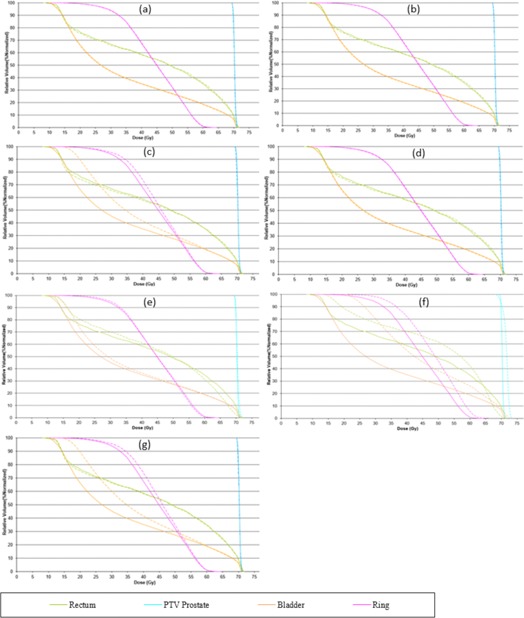
Patient B prostate plan. Dose‐volume histograms for the initial MF (solid lines) compared with (a) MF+0.5. (b) MF+1, (c) MF+2, (d) MF=5, (e) MF−0.5, (f) MF−1, and (g) MF, p=0.287 (dashed lines).

The plan for Patient B is shown in Fig. 2 and Table 5

**Figure 3:**
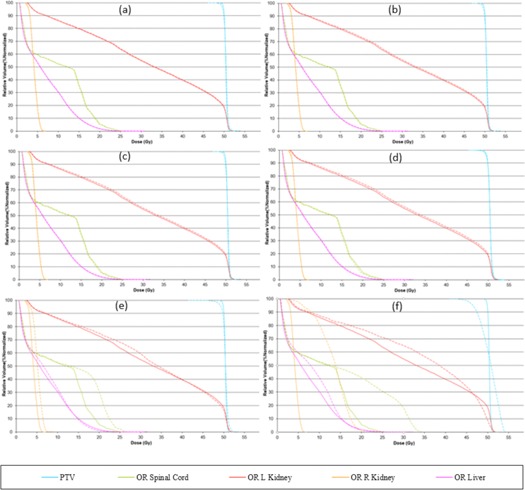
Patient C adrenal plan. Dose‐volume histograms for the initial MF (solid lines) compared with (a) MF+0.5. (b) MF+1, (c) MF+2, (d) MF=5, (e) MF−0.5, and (f) MF−1 (dashed lines).

Patient C plan (Fig. 3 and Table 6) was within the acceptable range of delivery quality assurance when replanned on AC and cheese phantom; however, modulation factors were still changed and replanned to verify the extent of delivery quality assurance.

**Figure 4:**
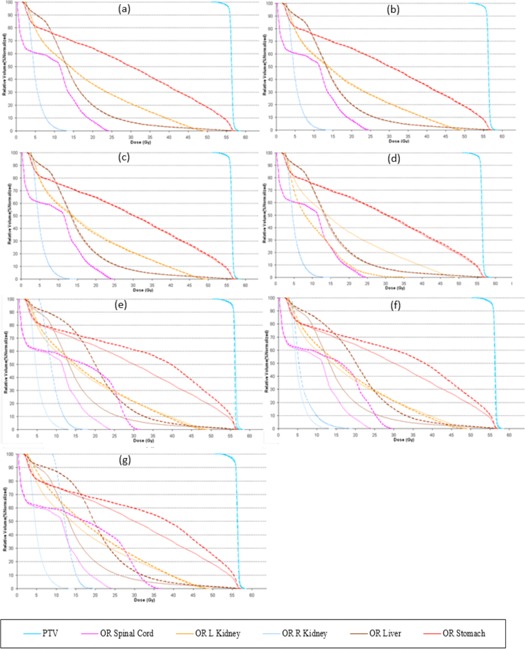
Patient D abdomen plan. Dose‐volume histograms for the initial MF (solid lines) compared with (a) MF+0.5. (b) MF+1, (c) MF+2, (d) MF=5, (e) MF−0.5, (f) MF−1, and (g) MF, p=0.430 (dashed lines).

Patient D (Fig. 4 and Table 7) showed no change in the uniformity of target coverage and OAR sparing however the plan deliverability was seen to improve slightly when the planned modulation factor was increased by 0.5, after which no improvements were seen in delivery. Reducing modulation factors did compromise the DVH constraints and the increased pitch penalized the OAR prescription.

**Figure 5:**
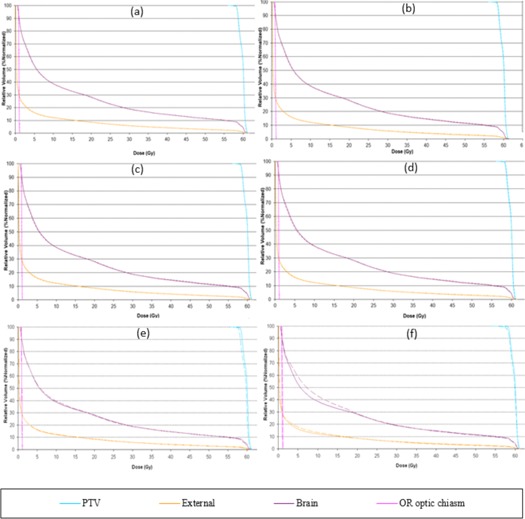
Patient D abdomen plan. Dose‐volume histograms for the initial MF (solid lines) compared with (a) MF+0.5. (b) MF+1, (c) MF+2, (d) MF=5, (e) MF−0.5, (f) MF−1, and (g) MF, p=0.430 (dashed lines).

The Patient E plan is shown in Fig. 5 and Table 8

**Figure 6:**
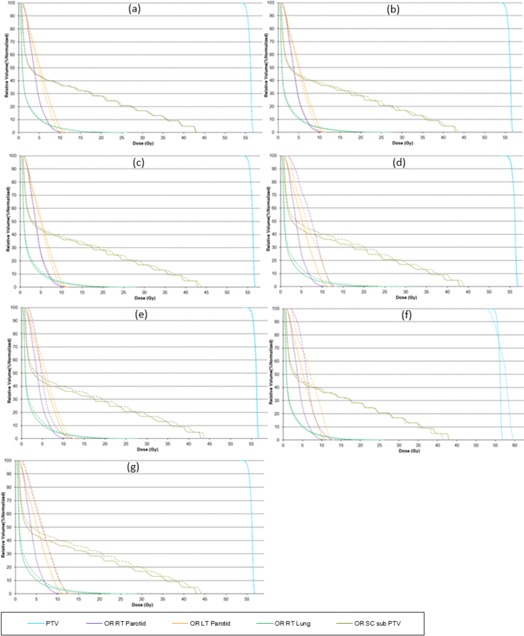
Patient F head and neck plan. Dose‐volume histograms for the initial MF (solid lines) compared with (a) MF+0.5, (b) MF+1, (c) MF+2, (d) MF=5, (e) MF−0.5, (f) MF−1, and (g) MF, p=0.287 (dashed lines).

In the case of Patient F (Fig. 6 and Table 9), it was observed that, due to the complexity of the treatment area (head and neck), achieving an optimized delivery plan was difficult as increasing the planned modulation factor did not improve the pass rate. However, reducing the MF by 0.5 did assist in achieving a better gamma pass rate (see Table 10). However decreasing the modulation factor any further did compromise PTV and OAR doses.

**Table 4:** Patient A uniformity index (UI), homogeneity index (HI), and OAR (%volume) for all replanned patient plans

			*OAR (% Vol)* ^a^		
*Patient A*	*PTV*		*Heart V25*	*RT Lung V20*	*LT Lung V20*
*Site: RT Breast*	*UI*	*HI*			
MF	1.12	0.12	14	38	0.2
MF+0.5	1.10	0.10	14	39	0.2
MF+1	1.11	0.10	14	38	0.2
MF+2	1.10	0.10	15	38	0.2
MF=5	1.10	0.10	15	38	0.2
MF−0.5	1.13	0.13	56	86	4.0
MF−1	1.12	0.12	56	83	4.3
SD	0.01	0.01			

a QUANTEC dose constraints: Heart−V25<10%,Lungs−V25<50%.

**Table 5:** Patient B uniformity index (UI), homogeneity index (HI), and OAR (%volume) for all replanned patient plans

			*OAR (% Vol)* ^a^	
*Patient B*	*PTV*		*Bladder V65*	*Rectum V50*
*Site: Prostate*	*UI*	*HI*		
MF	1.02	0.02	14	49
MF+0.5	1.02	0.02	13	49
MF+1	1.02	0.02	13	49
MF+2	1.02	0.02	13	48
MF=5	1.02	0.02	13	49
MF−0.5	1.03	0.03	14	49
MF−1	1.05	0.05	17	61
SD	0.01	0.01		

a QUANTEC dose constraints: Bladder−V65<50%,Rectum−V50<50%.

**Table 6:** Patient C uniformity index (UI), homogeneity index (HI), and OAR (%volume) for all replanned patient plans

			*OAR (% Vol)* ^a^
*Patient C*	*PTV*		*LT Kidney V12*
*Site: Adrenal*	*UI*	*HI*	
MF	1.09	0.09	34
MF+0.5	1.08	0.09	34
MF+1	1.07	0.08	34
MF+2	1.07	0.08	34
MF=5	1.07	0.08	23
MF−0.5	1.14	0.13	38
MF−1	1.21	0.20	40
SD	0.05	0.05	

a QUANTEC dose constraints: Kidney−V12<55%.

**Table 7:** Patient D uniformity index (UI), homogeneity index (HI), and OAR (%volume) for all replanned patient plans

			*OAR (% Vol)* ^a^	
*Patient D*	*PTV*		*LT Kidney V12*	*Liver V30*
*Site: Abdomen*	*UI*	*HI*		
MF	1.07	0.07	55	6.9
MF+0.5	1.07	0.07	55	6.9
MF+1	1.07	0.07	54	7.0
MF+2	1.07	0.08	54	7.0
MF=5	1.07	0.08	54	7.1
MF−0.5	1.07	0.07	57	14
MF−1	1.08	0.08	59	15
SD	0.00	0.00		

a QUANTEC dose constraints: Kidney−V12<55%,Liver−V30<50%.

**Table 8:** Patient E uniformity index (UI), homogeneity index (HI), and OAR (%volume) for all replanned patient plans

			*OAR (Max Dose)* ^a^
*Patient E*	*PTV*		*Brainstem Max dose*
*Site: Brain*	*UI*	*HI*	
MF	1.06	0.07	5.8
MF+0.5	1.05	0.05	5.7
MF+1	1.05	0.05	5.7
MF+2	1.05	0.05	5.5
MF=5	1.04	0.05	5.5
MF−0.5	1.07	0.07	5.8
MF−1	1.08	0.09	6.0
SD	0.01	0.02	

a QUANTEC dose constraints: Brainstem Max dose <54Gy.

**Table 9:** Patient F uniformity index (UI), homogeneity index (HI), and OAR (%volume) for all replanned patient plans

			*OAR (Max Dose)* ^a^		
*Patient F*	*PTV*		*SC subPTV V45*	*LT Parotid V25*	*RT Parotid V25*
*Site: Head and Neck*	*UI*	*HI*			
MF	1.04	0.05	43	12	10
MF+0.5	1.04	0.05	43	12	11
MF+1	1.04	0.04	44	12	11
MF+2	1.04	0.04	44	12	11
MF=5	1.05	0.05	44	14	13
MF−0.5	1.05	0.05	44	12	13
MF−1	1.11	0.11	41	12	13
SD	0.02	0.02			

a QUANTEC dose constraints: SC subPTV−V45<50%,Parotid−V25<20%.

**Table 10:** Fluence gamma results using the AC diode array using replanned patient plans

*Treatment Site*	*Pat*.	*MF*	MF+0.5	MF+1	MF+2	MF=5	MF−0.5	MF−1
RT Breast	A	95	98	99	98	96	92	88
Prostate	B	68	96	99	98	83	88	86
Adrenal	C	98	98	75	78	76	98	97
Abdomen	D	78	89	68	63	60	67	61
Brain	E	89	98	75	61	57	89	84
Head and Neck	F	74	56	71	64	46	92	86

### C. Replanned point dose and fluence measurements

From 3, 10, 11 it was concluded that actual modulation factors between 1.4 and 2.5 contributed towards a clinically acceptable plan and passed delivery quality assurance. However, factors like dose coverage and treatment times are also important parameters to consider.

Table 12 shows that the change in pitch for the same modulation factor does not improve the plan substantially, but can deviate from the actual planning goal during optimization, as seen in the DVH analysis section. However, while increasing modulation factors, the pitch is also decreased to suit optimization constraints.

**Table 11:** Point‐dose percentage error for all replanned patient plans

*Treatment Site*	*Pat*.	*MF*	MF+0.5	MF+1	MF+2	MF=5	MF−0.5	MF−1
RT Breast	A	1.1	1.3	1.7	1.6	2.2	−3.3	−2.5
Prostate	B	−1.6	−2.0	−1.0	0.0	−1.7	−1.5	−1.3
Adrenal	C	1.8	1.7	1.9	1.4	2.1	−1.2	0.8
Abdomen	D	1.7	1.5	1.7	2.6	2.8	−2.0	−2.3
Brain	E	0.9	−2.4	−1.9	−1.9	0.3	−1.4	−2.6
Head and Neck	F	−1.4	−1.3	−0.3	1.1	0.6	−2.0	−1.2

**Table 12:** Fluence and point‐dose measurements for changed pitch plans

*Treatment Site*	*Pat*.	*MF*	*Initial Pitch*	*Changed Pitch*	*Gamma Fluence*		*Point Dose % error*	
					*Before*	*After*	*Before*	*After*
Prostate	B	2.2	0.430	0.287	68	85	−1.6	−3.9
Abdomen	D	2.5	0.287	0.43	78	58	1.7	−0.8
Head and Neck	F	2.2	0.430	0.287	74	54	−1.4	−1.1

### D. Planned leaf sinograms and LOTs

**Figure 7:**
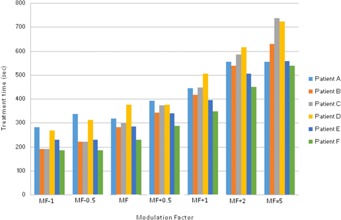
Modulation factor vs. treatment times (s) for all patient plans.

As discussed earlier, planned leaf sinograms show projection intensity verses leaf number for a particular modulation factor and pitch value. As the modulation factor is increased it can be seen that the intensity of the projection is reduced, making the treatment time longer, and the delivery deviates from the intended plan, and this is the same when plans have a reduced modulation factor beyond a certain limit. Delivery pass criteria were met when the modulation factor was within the range 1.4–2.5. This work illustrates that the use of very fast or slow leaf open times can exceed the machine limitations of leaf motions, which can translate into inaccuracies between a plan and its delivery. Leaf latencies are modeled by the vendor on the assumption that the relationship between the planned and actual leaf open times is linear.^(1)^ Leaf latencies represent the differences between the planned and actual leaf open times. However, it was observed that, by increasing the mean leaf open times, treatment times were higher and delivery pass rates were reduced (see Table 13 and Fig. 7).

**Figure 8:**
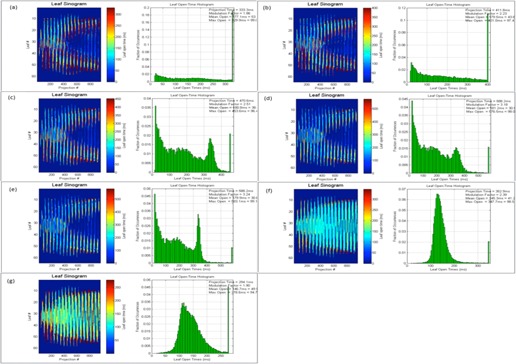
Patient ART breast plan. Leaf sinograms and LOT: (a) initial MF, (b) MF+0.5, (c) MF+1, (d) MF+2, (e) MF=5, (f) MF−0.5, and (g) MF−1.

**Figure 9:**
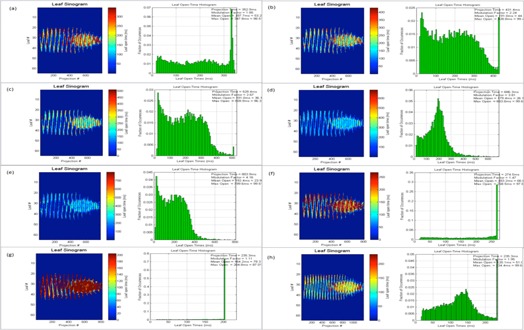
Patient B prostate plan. Leaf sinograms and LOT: (a) initial MF, (b) MF+0.5, (c) MF+1, (d) MF+2, (e) MF=5, (f) MF−0.5, (g) MF−1, and (h) p=0.287.

**Figure 10:**
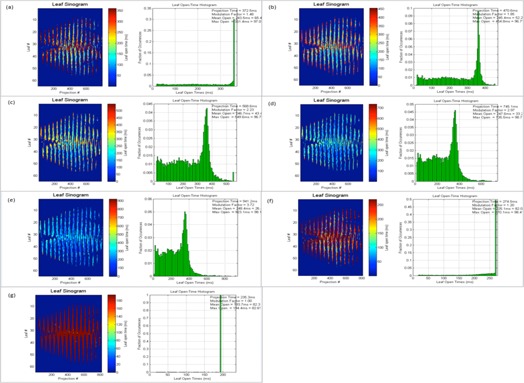
Patient C adrenal plan. Leaf sinograms and LOT: (a) initial MF, (b) MF+0.5, (c) MF+1, (d) MF+2, (e) MF=5, (f) MF−0.5, and (g) MF−1.

**Figure 11:**
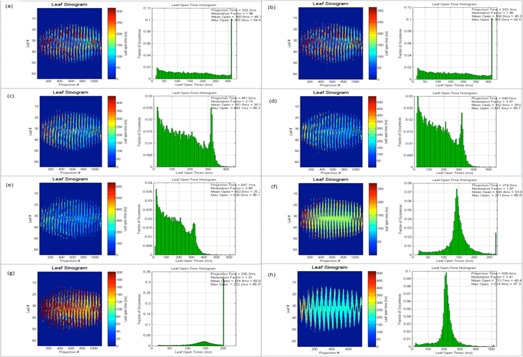
Patient D abdomen plan. Leaf sinograms and LOT: (a) initial MF, (b) MF+0.5, (c) MF+1, (d) MF+2, (e) MF=5, (f) MF−0.5, (g) MF−1, and (h) p=0.430.

**Figure 12:**
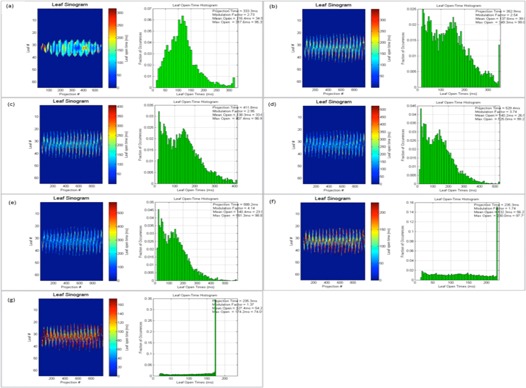
Patient E brain plan. Leaf sinograms and LOT: (a) initial MF, (b) MF+0.5, (c) MF+1, (d) MF+2, (e) MF=5, (f) MF−0.5, and (g) MF−1.

**Figure 13:**
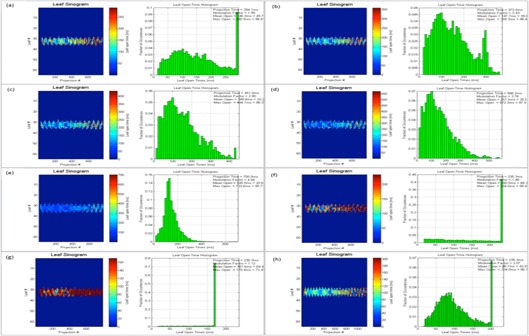
Patient F head and neck plan. Leaf sinograms and LOT: (a) initial MF, (b) MF+0.5, (c) MF+1, (d) MF+2, (e) MF=5, (f) MF−0.5, (g) MF−1, and (h) p=0.287.

The following figures show the plan results for right breast (Patient A, Fig. 8), prostate (Patient B, Fig. 9), adrenal gland (Patient C, Fig. 10), abdomen (Patient D, Fig. 11), brain (Patient E, Fig. 12), and head and neck (Patient F, Fig. 13).

**Table 13:** Gantry rotation period (s) for all patient plans

*Treatment Site*	*Pat*.	*MF*	MF+0.5	MF+1	MF+2	MF=5	MF−0.5	MF−1	p=0.287	p=430
RT Breast	*A*	17	21	24	30	30	18	15	–	–
Prostate	B	18	22	27	35	41	14	12	12	–
Adrenal	C	19	24	29	38	48	14	12	–	–
Abdomen	D	17	17	23	28	33	14	12	–	27
Brain	E	17	18	21	27	30	12	12	–	–
Head and Neck	F	15	19	23	30	36	12	12	–	12

## IV. DISCUSSION

In this study, the range of actual modulation factors that achieved an acceptable delivery pass was seen to be within a band of 1.4 to 2.5. This indicates that an optimized plan with delivery acceptability can be possible with smaller leaf open times. However, very small leaf open times can cause inaccuracies in the MLC latencies, thereby making the plan unachievable. The optimizer may also struggle to achieve a planned goal as the reduced time constraint can affect the dosimetric aspect of the treatment. As seen in 8, 9, 10, 11, 12, 13, increasing this mean open time has caused a larger proportion of the planned MLCs to open before the 100 ms and this could be a cause that affects machine capabilities. Reducing the modulation factor has consistently kept the average mean leaf open times smaller, whereas the larger modulation factors caused higher treatment delivery time (as shown in 4, 5, 6, 7, 8, 9). The use of varied pitch values for the three plans considered haven't improved the measured fluence pass criteria. Point‐dose measurements are seen to be greater than the planned measurements (see 11, 12) for plans optimized using the greatest modulation factor of 5. In case of plans with reduced modulation factors, it can be seen that the measured dose is generally lower to the planned dose value. This could be due to the planning system not being able to fully accommodate lower modulation in its actual fluence calculations. According to the fluence results in this study (see Table 11), the use of the greatest modulation factor has made no significant change in the DVHs, but has compromised plan delivery and is, therefore, not recommended. Leaf sinograms for all patient plans can be used as a guide to assess delivery quality — as the intensity of the projection reaches either a maximum or a minimum intensity, plan deliverability is harder to achieve (see 7, 8, 9, 10, 11, 12). The vendors have now reported that by introducing non‐integer gantry rotation period the delivery discrepancies can be fixed for reduced MF plans. This is a future work for systems enabled with this feature as the current study involved integer gantry rotation period that was available during the study.

## V. CONCLUSIONS

From a previous study^(1)^ it was concluded that increasing mean leaf opening times for failed plans in small increments may improve delivery accuracy. This study looked at the actual modulation factors in plans across a range of treatment sites and it was observed that the range of modulation factors used by the TPS between 1.4 and 2.5 fall within the optimal scope of passing delivery quality assurance for most plans. However, plans that fall in this range can also fail DQA analysis due to the nonlinear planned and actual delivery fluence relationship caused by machine limitations. This discrepancy can be further resolved by increasing or decreasing the upper limits of maximum to mean leaf open times by integral increments. Increasing modulation factors increase treatment times as well, therefore care must be taken during replan to ensure the organ at risk constraints are not exceeded. The modulation and pitch factor also contribute to the gantry period of rotation and, therefore, MLC leaf latencies can be reduced by keeping its minimum value to 15 s. DVHs for all patient plans show that increasing modulation factors does not change the uniformity of target or OAR dose limits substantially; however, reducing the same can impact plan quality. Therefore, using an optimized modulation factor with a good pitch can ensure less treatment time with maximum delivery accuracy.

## ACKNOWLEDGMENTS

The authors would like to thank Jong Gi Lee from TomoTherapy Inc. and Lukasz Wlodarczyk from Sun Nuclear Corporation for their technical support and advice during the measurement analysis of this investigation. The authors would also like to extend their gratitude towards all tomotherapy planners at the RBWH Radiation Therapy department for their contribution during the discussions of this study and ongoing support.
